# Supplementary material for: Measurement invariance of the Center for Epidemiological Studies-Depression scale and associations with genetic risk in older adults
Source: PLoS One. 2024 Oct 28;19(10):e0312194. doi: 10.1371/journal.pone.0312194 (PMC11515990; doi:10.1371/journal.pone.0312194)
Supplement: S1 File — (DOCX) [file pone.0312194.s001.docx]

# Additional information about participants and methods

## Participants

This study used two sub-cohorts from the older Finnish Twin Cohort (FTC) study [1]. The first and the larger sub-cohort comprised 1698 individuals from MEMTWIN II. The MEMTWIN II sub-study focused on cognitive screening instruments in older adults, but also depressive symptom data and saliva samples were collected. MEMTWIN II data were collected from twins born between 1938–1944, and even those without a co-twin were asked to participate in the study.

The MEMTWIN II cohort was split into exploratory and confirmatory samples by requiring distinct family numbers. This method assigns the smaller ID number to the exploratory sample and larger to the confirmatory sample leading to twins being in separate groups. Of note, the ID numbers do not reflect birth order or the time of assessment.

The NONAGINTA – Memory and health in 90-year-olds study is an ongoing sub-study of the older FTC participants who are 90 years old or turn 90 during the study data collection. NONAGINTA study protocol includes cognitive phone interview, saliva sample collection and postal questionnaire about health and health-related behavior including self-reported depressive symptoms as measured with the 20-item CES-D. To date, we have invited twins who were born in year 1932 or earlier to participate in NONAGINTA. We invite all twins irrespectively of their co-twin’s vital status to participate. All participants are from same-sex twin pairs.

## Telephone Interview for Cognitive Status-modified

For cognitive assessment the modified Telephone Interview for Cognitive Status (TICS-m) [2] was used. TICS-m is a 50-point telephone-administered cognitive screening test with the following items: name (2 points), age (1 point), telephone number (1 point), date (5 points), current president (2 points), previous president (this item is “vice president” in the original, but was changed for the Finnish version due to absence of a vice president in Finland; 2 points), counting backwards (2 points), immediate recall of a 10-word list (10 points), subtracting sevens (5 points), responsive naming (4 points), repetition of phrases (2 points), finger tapping (2 points) and delayed recall of the 10-word list (10 points). The TICS-m procedure has been described in detail in Lindgren et al.[2] To accommodate for low scores in individuals with low education, we used the previously published education adjustment for the total score of the TICS-m [3] with a cut-off of ≤ 31 for cognitive impairment (≤ 27 is the recommended cut-off for dementia and scores 28-31 indicate mild cognitive impairment). For individuals with ≤8 years of education, 5 points are added to the total score; 2 points are added for individuals with 8-10 years of education; the total score is not adjusted for individuals with 11-15 years of education and 2 points are subtracted from individuals with ≥16 years of education. As a cognitive screening instrument, the TICS-m is not equivalent to a complete neuropsychological assessment and it may fail to detect subtle cognitive difficulties in those classified as cognitively normal. The mean years of education in the MEMTWIN II sample was 10.3 (SD 3.9), see Lindgren et al. [2] for details.

## Examination of response styles and missing data

To assess for the presence of biased data due to response styles, the CES-D score distributions were examined before reverse-coding. Distributions of missing CES-D data were analyzed between groups (cognitively unimpaired versus cognitively impaired; MEMTWIN II versus NONAGINTA). Individuals with complete CES-D data without data required for education-adjusted TICS-m total score (TICS-m total score and/or education were missing) were compared to those with complete TICS-m total score data and education in terms of sex, age and CES-D total score.

## Factor model estimation and reporting

Kaiser-Meyer-Olkin coefficient of sampling adequacy and Bartlett’s test of sphericity were run, and communalities examined prior to exploratory factor analyses. Parallel analysis was used to determine the number of factors to be retained [4,5]. We used minimum residuals as our method of factor extraction. We used oblimin rotation as oblique solutions may not diverge from orthogonal rotations even in the case of non-correlated factors, whereas the opposite is not true [6].

For confirmatory factor analyses, diagonal weighted least squares (DWLS) was used as the estimator and Root Mean Square Error of Approximation (RMSEA) [7], standardized root mean square residual (SRMR), Tucker-Lewis Index (TLI) [8], and comparative fit index (CFI) [9] were reported as fit indices. RMSEA <. 06 was considered to indicate good fit, <. 08 fair fit and < .10 mediocre fit [7,10,11]; SRMR < .08, CFI and TLI >. 95 also indicated good fit [10]. For reliability coefficients, we reported Cronbach’s α for convention and omega total (ω_T_) as a more robust option. There are limitations to Cronbach’s α [12,13] and other reliability coefficients are increasingly preferred, such as ω_T_ [14].

As described in the main text, polychoric correlations were used in model estimation. However, polychoric correlations were not used for the estimation of reliability coefficients, as this has been considered to artificially inflate the coefficients [15].

## Factor analyses using ordinal data

Many previous structural examinations of the CES-D and its shorter forms have used principal components analysis and/or treated the ordinal item data as continuous. Both practices may have led to biased factor solutions [5,16,17], which we have attempted to avoid by using appropriate factoring methods and treating the data as ordinal.

The traditional cut-offs used in evaluating the fit of CFA are based on simulation studies using maximum likelihood estimation [10]. DWLS estimation was used in this study and it is appropriate for ordinal data [16], but this estimation method is prone to finding better fit indices compared to maximum likelihood estimation and the usefulness of conventional, maximum likelihood estimation-based criteria for evaluating model fit is unclear [18,10]. This could particularly be the case for the multi-factor models, which have factors with 2 indicators. Generally, 3 to 5 indicators per factor is considered a minimum sufficient ratio [5], but the practical consequences of conventional CES-D models having 2-indicator factors is not often discussed.

## Sum scores versus factor scores

With factor analyses, an opportunity is presented to use factor scores in further analyses in the data. Such is the case in this study for the polygenic risk score associations. There are proponents for using factor scores over unit-weighted sum scores (meaning the scores of the used scales are not transformed in any way) whenever possible [19]. However, since factor scores are reliant on the choice of the factoring method, the choice of the factor score regression or structural equation modelling method and the properties of the sample in question [13,20], factor scores are hardly comparable across studies. Thus, in the trade-off between using factor scores for possibly more accurate representation of the depression constructs under investigation in this study versus generalizability to other studies using sum scores [21], we opted for the latter [13,19].

## References

1. Kaprio J, Bollepalli S, Buchwald J, Iso-Markku P, Korhonen T, Kovanen V, et al. The Older Finnish Twin Cohort — 45 Years of Follow-up. Twin Res Hum Genet. 2019 Aug;22(4):240–54.

2. Lindgren N, Rinne JO, Palviainen T, Kaprio J, Vuoksimaa E. Prevalence and correlates of dementia and mild cognitive impairment classified with different versions of the modified Telephone Interview for Cognitive Status (TICS‐m). Int J Geriatr Psychiatry. 2019 Dec;34(12):1883–91.

3. Knopman DS, Roberts RO, Geda YE, Pankratz VS, Christianson TJH, Petersen RC, et al. Validation of the Telephone Interview for Cognitive Status-modified in Subjects with Normal Cognition, Mild Cognitive Impairment, or Dementia. Neuroepidemiology. 2010;34(1):34–42.

4. Baglin J. Improving Your Exploratory Factor Analysis for Ordinal Data: A Demonstration Using FACTOR. Pract Assess Res Eval. 2014;19:1–15.

5. Fabrigar LR, Wegener DT, MacCallum RC, Strahan EJ. Evaluating the Use of Exploratory Factor Analysis in Psychological Research. Psychol Methods. 1999;4(3):272–99.

6. Osborne JW. What is Rotating in Exploratory Factor Analysis? Pract Assess Res Eval. 2015;20:1–7.

7. Browne MW, Cudeck R. Alternative Ways of Assessing Model Fit. Sociol Methods Res. 1992 Nov;21(2):230–58.

8. Tucker LR, Lewis C. A reliability coefficient for maximum likelihood factor analysis. Psychometrika. 1973 Mar;38(1):1–10.

9. Bentler PM. Comparative fit indexes in structural equation model. Psychol Bull. 1990;107(2):238–46.

10. Hu L, Bentler PM. Cutoff criteria for fit indexes in covariance structure analysis: Conventional criteria versus new alternatives. Struct Equ Model Multidiscip J. 1999 Jan;6(1):1–55.

11. MacCallum RC, Widaman KF, Zhang S, Hong S. Sample size in factor analysis. Psychol Methods. 1999;4(1):84–99.

12. Cortina JM. What is coefficient alpha? An examination of theory and applications. J Appl Psychol. 1993;78(1):98–104.

13. Widaman KF, Revelle W. Thinking thrice about sum scores, and then some more about measurement and analysis. Behav Res Methods [Internet]. 2022 Apr 25 [cited 2023 Mar 7]; Available from: https://link.springer.com/10.3758/s13428-022-01849-w

14. McNeish D. Thanks Coefficient Alpha, We’ll Take It From Here. Psychol Methods. 2017;23(3):1–23.

15. Revelle W, Condon DM. Reliability from α to ω: A tutorial. Psychol Assess. 2019 Dec;31(12):1395–411.

16. Li CH. The performance of ML, DWLS, and ULS estimation with robust corrections in structural equation models with ordinal variables. Psychol Methods. 2016 Sep;21(3):369–87.

17. Rhemtulla M, Brosseau-Liard PÉ, Savalei V. When can categorical variables be treated as continuous? A comparison of robust continuous and categorical SEM estimation methods under suboptimal conditions. Psychol Methods. 2012 Sep;17(3):354–73.

18. Xia Y, Yang Y. RMSEA, CFI, and TLI in structural equation modeling with ordered categorical data: The story they tell depends on the estimation methods. Behav Res Methods. 2019 Feb;51(1):409–28.

19. McNeish D, Wolf MG. Thinking twice about sum scores. Behav Res Methods. 2020 Dec;52(6):2287–305.

20. van Bork R. Interpreting psychometric models. University of Amsterdam; 2019.

21. Frank P, Ajnakina O, Steptoe A, Cadar D. Genetic susceptibility, inflammation and specific types of depressive symptoms: evidence from the English Longitudinal Study of Ageing. Transl Psychiatry. 2020 May 12;10(1):140.
