# Supplementary material for: Measurement invariance of the Center for Epidemiological Studies-Depression scale and associations with genetic risk in older adults
Source: PLoS One. 2024 Oct 28;19(10):e0312194. doi: 10.1371/journal.pone.0312194 (PMC11515990; doi:10.1371/journal.pone.0312194)
Supplement: S2 File — (DOCX) [file pone.0312194.s002.docx]

## **Missing values and response style**

CES-D score distribution analyses revealed that 5 (0.3%) participants in the MEMTWIN II cohort and 1 participant (0.7%) in the NONAGINTA cohort had extreme values before reverse-coding of the items, indicating potential extreme response style bias. 48 participants of the MEMTWIN II sample had incomplete data to calculate the education-adjusted TICS-m total score (n=45 missing TICS-m total score, n=3 missing education). These participants with incomplete TICS-m data had higher CES-D total scores (*t*(48)= 3.71, p < .001), but did not differ from those with full TICS-m data in terms of education *t*(48)= -2, p = 0.05) or sex (χ^2^[1] = 0.51, p = 0.47). Participants with missing TICS-m data tended to also have missing age data, precluding formal testing for age differences between those with and without TICS-m data.
