# Supplementary material for: Measurement invariance of the Center for Epidemiological Studies-Depression scale and associations with genetic risk in older adults
Source: PLoS One. 2024 Oct 28;19(10):e0312194. doi: 10.1371/journal.pone.0312194 (PMC11515990; doi:10.1371/journal.pone.0312194)
Supplement: S1 Table — (DOCX) [file pone.0312194.s003.docx]

| Supplementary Table 1. Correlations among factors in multi-factor models. | | | | | | | | |
| --- | --- | --- | --- | --- | --- | --- | --- | --- |
|  | CES-D 20 | | | |  | CES-D 8 | | |
|  | Factor 1 | Factor 2 | Factor 3 | Factor 4 |  | Factor 1 | Factor 2 | Factor 3 |
| Factor 1 | - |  |  |  |  | - |  |  |
| Factor 2 | 0.872 | - |  |  |  | 0.697 | - |  |
| Factor 3 | 0.724 | 0.681 | - |  |  | 0.789 | 0.548 | - |
| Factor 4 | 0.653 | 0.644 | 0.613 | - |  |  |  |  |
