# Supplementary material for: Measurement invariance of the Center for Epidemiological Studies-Depression scale and associations with genetic risk in older adults
Source: PLoS One. 2024 Oct 28;19(10):e0312194. doi: 10.1371/journal.pone.0312194 (PMC11515990; doi:10.1371/journal.pone.0312194)
Supplement: S2 Table — (DOCX) [file pone.0312194.s004.docx]

| Supplementary Table 2. CES-D score differences between those with cognitive impairment and those with normal cognition in the MEMTWIN II cohort. | | | | | | | |
| --- | --- | --- | --- | --- | --- | --- | --- |
|  | Cognitively impaired |  | Cognitively unimpaired |  | Difference, β | 95% CI Lower | 95% CI Upper |
| n | 260 |  | 1390 |  |  |  |  |
| CES-D 20, M (SD) | 7.9 (7.8) |  | 7.5 (7.1) |  | -0.49 | -1.09 | 0.11 |
| CES-D 15, M (SD) | 4.9 (5.7) |  | 4.6 (5.1) |  | -0.35 | -0.83 | 0.12 |
| CES-D 8, M (SD) | 4 (3.9) |  | 3.7 (3.6) |  | -0.24 | -0.52 | 0.03 |
| Difference is standardized regression coefficient for the grouping variable (reference = cognitively unimpaired), with CES-D scores as dependent variables and cognitive status, sex and age as independent variables and clustered standard errors for twin structures. | | | | | | | |
