# Supplementary material for: Measurement invariance of the Center for Epidemiological Studies-Depression scale and associations with genetic risk in older adults
Source: PLoS One. 2024 Oct 28;19(10):e0312194. doi: 10.1371/journal.pone.0312194 (PMC11515990; doi:10.1371/journal.pone.0312194)
Supplement: Attachment — Submitted filename: Peer Review.docx [file pone.0312194.s005.docx]

**Peer Review “Measurement invariance of the Center for Epidemiological Studies-Depression scale and associations with genetic risk in older adults”**

The manuscript aims to propose that different versions of the CES-D are capable of measuring depressive symptoms, particularly in individuals over the age of 90 and/or those with cognitive impairment. Higher MDD-PRS (Major Depressive Disorder Polygenic Risk Score) was associated with more depressive symptoms across various CES-D versions.

Moreover, the authors suggest that the CES-D maintains consistent constructs in the presence or absence of cognitive impairment and among individuals over 90 years old as well as those in their 70s, even with a higher polygenic risk score associated with elevated CES-D scores.

**Strengths:**

- The exploratory and confirmatory factor analyses are thorough and consistent; the information and supporting results are clear.
- The results are presented in a clear and didactic manner.

***Major Issues***

- The results related to the association between genetic risk for depression and CES – D scores, one of the aims of the study, could be explore further

**Minor issues**

- It’s no clear how in this study were controlled strange variables, such as anosognosia effect in self report
- The authors don’t include graphs particularly in the Results section (for example, these could be usefull in exploratory and confirmatory factor analyses sumarize)
- Explore further the associations with genetic risk for depression in the results.
