# Supplementary material for: Measurement invariance of the Center for Epidemiological Studies-Depression scale and associations with genetic risk in older adults
Source: PLoS One. 2024 Oct 28;19(10):e0312194. doi: 10.1371/journal.pone.0312194 (PMC11515990; doi:10.1371/journal.pone.0312194)
Supplement: Attachment — Submitted filename: Response letter.docx [file pone.0312194.s006.docx]

We thank the reviewers and the editor for their encouraging and insightful comments which have improved the manuscript. The changes in the manuscript are noted by Tracked Changes and by page (P#) in italicized text in this response letter. The responses are listed in the following order: comments by the editor, comments by the reviewers in a separate document, and comments by the reviewers included in the manuscript decision message.

Editor’s Comments

**1.** Please ensure that your manuscript meets PLOS ONE's style requirements, including those for file naming. The PLOS ONE style templates can be found at <https://journals.plos.org/plosone/s/file?id=wjVg/PLOSOne_formatting_sample_main_body.pdf>  and 
<https://journals.plos.org/plosone/s/file?id=ba62/PLOSOne_formatting_sample_title_authors_affiliations.pdf>

**Response:** We have ensured that our manuscript meets the journal’s style requirements.

**2.** Thank you for stating the following financial disclosure: 
"Data collection and analyses in the Finnish Twin Cohort have been supported by the Academy of Finland (grants to JK: 312073, 336823, 352792). MEMTWIN II data collection was supported by the Sigrid Juselius foundation, The Academy of Finland (grants 133193 and 310962) and Finnish Governmental Research Funding (VTR). NONAGINTA study was funded by the Academy of Finland grants (320109 and 345988 to EV). EV was supported by the Academy Research Fellow grant 314639 and the Sigrid Jusélius Foundation. Open access funded by Helsinki University Library."

Please state what role the funders took in the study.  If the funders had no role, please state: ""The funders had no role in study design, data collection and analysis, decision to publish, or preparation of the manuscript."" 
If this statement is not correct you must amend it as needed. 
Please include this amended Role of Funder statement in your cover letter; we will change the online submission form on your behalf.

**Response:** Funders had no role in the study and the corresponding sentence has been added to the financial disclosure.

**3.** Thank you for stating the following in the Competing Interests section: 
"JK has received support from Sigrid Jusélius Foundation, the European Union and the National Institutes of Health. JOR has consulted for Clinical Research Services Turku and acted as a member on the data monitoring committee for Lundbeck."

Please confirm that this does not alter your adherence to all PLOS ONE policies on sharing data and materials, by including the following statement: ""This does not alter our adherence to  PLOS ONE policies on sharing data and materials.” (as detailed online in our guide for authors <http://journals.plos.org/plosone/s/competing-interests>).  If there are restrictions on sharing of data and/or materials, please state these. Please note that we cannot proceed with consideration of your article until this information has been declared.

Please include your updated Competing Interests statement in your cover letter; we will change the online submission form on your behalf.

**Response:** The updated Competing Interests statement has been added to the cover letter as requested: “This does not alter our adherence to PLOS ONE policies on sharing data and materials.”.

**4.** Please review your reference list to ensure that it is complete and correct. If you have cited papers that have been retracted, please include the rationale for doing so in the manuscript text, or remove these references and replace them with relevant current references. Any changes to the reference list should be mentioned in the rebuttal letter that accompanies your revised manuscript. If you need to cite a retracted article, indicate the article’s retracted status in the References list and also include a citation and full reference for the retraction notice.

**Response:** We have reviewed the reference list for completeness and correctness. None of the cited papers have been retracted.

Reviewer comments

**Peer Review “Measurement invariance of the Center for Epidemiological Studies-Depression scale and associations with genetic risk in older adults”**

The manuscript aims to propose that different versions of the CES-D are capable of measuring depressive symptoms, particularly in individuals over the age of 90 and/or those with cognitive impairment. Higher MDD-PRS (Major Depressive Disorder Polygenic Risk Score) was associated with more depressive symptoms across various CES-D versions.

Moreover, the authors suggest that the CES-D maintains consistent constructs in the presence or absence of cognitive impairment and among individuals over 90 years old as well as those in their 70s, even with a higher polygenic risk score associated with elevated CES-D scores.

**Strengths:**

- The exploratory and confirmatory factor analyses are thorough and consistent; the information and supporting results are clear.
- The results are presented in a clear and didactic manner.

***Major Issues***

**1.** The results related to the association between genetic risk for depression and CES – D scores, one of the aims of the study, could be explore further

**Response:** We attempted to be exhaustive in our analyses by looking at all CES-D scales and subscales in the regression analyses with MDD-PRS. The only addition we could think of was to examine the CES-D 20, 15 and 8 scores for each MDD-PRS decile. Thus, we added Figure 2 to show these distributions.

P15: *“In MDD-PRS decile plots (Fig2), the differences between different deciles on CES-D scores were quite low, but CES-D scores were the highest in the top decile for MDD-PRS.”*

**Minor issues**

**2.** It’s no clear how in this study were controlled strange variables, such as anosognosia effect in self report

**Response:** Anosognosia can indeed affect the validity of self-report measures, including CES-D. We have added the following text to Discussion:

P17: *“It is also possible that anosognosia could have affected the scores of individuals with cognitive impairment. Therefore, not adjusting for anosognosia can be considered a limitation of the study.”*

**3.** The authors don’t include graphs particularly in the Results section (for example, these could be usefull in exploratory and confirmatory factor analyses sumarize)

**Response:** We agree with the reviewer that the results rely heavily on tables and text. We believe that the presentation of factor analyses as tables follows the conventional reporting of these analyses, especially with a considerable number of models (exploratory and confirmatory, measurement invariance) and is appropriate as such. However, we do agree with the reviewer that visualization can be helpful and have made a few additions to the manuscript. First, we added Fig1 to show how the MEMTWIN II sample was divided for exploratory and confirmatory analyses. Second, to display more information about the relationship between MDD-PRS and CES-D, we have added a panel of decile plots showing the CES-D scores for each decile of the MDD-PRS (Fig2).

**4.** Explore further the associations with genetic risk for depression in the results.

**Response:** Please see our response to the comment 1.

Additional Reviewer Comments:

**5.** Reviewer #1: The manuscript has a logical and sound structure and methodological strengths, and it is written in standard, correct, and clear English.

As a suggestion, the statistical analyses should be confirmed.

**Response:** We apologize, but it is not entirely clear to us what the referee means by “confirming statistical analyses” here. Please note, that we have provided code for the analyses which can be modified to be used in other data sets for similar data.

**6.** Reviewer #2: To improve the clarity of the materials and methods section, it would be appropriate to add a figure of the participants in the study and to subdivide the measures section into subsections to improve its understanding by the reader to explain the instruments in more detail. Additionally, it is necessary to review the writing of the statistical analysis section to make it clearer to the reader.

**Response:** We have now divided the measures section into three subdivisions, namely CES-D, TICS-m and polygenic risk score. The full description of TICS-m is lengthy, which is why the detailed description of the measure is shown in the Supplementary File 1. Figure 1 has been added to show how the models were ran in the subsamples of MEMTWIN II.

P7: “*Fig1 shows which analyses were conducted in each subsample of MEMTWIN II.”*

This Figure is now also referenced in the paragraph concerning measurement invariance testing. We hope that this figure helps the reader in conceptualizing which models were used in the measurement invariance part of the paper.

P8-9: *“These four invariance models were tested with the CES-D 20 and CES-D 8 models derived from our exploratory analyses and the two theory-driven models – CES-D 15 [10] and CES-D 8 [11] with correlated residuals – mentioned previously (structures from Fig1 confirmatory analyses).”*

In the manuscript, we have tried to be as precise as possible about the modelling decisions and added supplementary text to complement the main text. We did also provide a link to the R code for running the analyses, which can be checked by the reader of this paper as verbal descriptions of analyses are often ambiguous. Despite these efforts, we understand that some parts of the text can still be unclear. We are happy to clarify any specific parts of the statistical analyses that the reviewer considers particularly problematic to follow.
